# The effect of N-acetyl cysteine (NAC) on aluminum phosphide poisoning inducing cardiovascular toxicity: a case–control study

**DOI:** 10.1186/s40064-016-3630-2

**Published:** 2016-11-10

**Authors:** Fakhreddin Taghaddosinejad, Esmaeil Farzaneh, Mahdi Ghazanfari-Nasrabad, Nastaran Eizadi-Mood, Morteza Hajihosseini, Omid Mehrpour

**Affiliations:** 1 Department of Forensic Medicine, Tehran University of Medical Sciences, Tehran, Iran; 2 Department of Forensic Medicine and Toxicology, Ardabil University of Medical Sciences, Ardabil, Iran; 3 Legal Medicine Research Center, Legal Medicine Organization, Bahar Cross, Taleghani Avenue, Tehran, 1114795113 Iran; 4 Isfahan Clinical Toxicology Research Center, Isfahan University of Medical Sciences, Isfahan, Iran; 5 Birjand CardioVascular Diseases Research Center, Birjand University of Medical Sciences, Moallem Avenue, Birjand, 9713643138 Iran; 6 Medical Toxicology and Drug Abuse Research Center (MTDRC), Birjand University of Medical Sciences, Moallem Avenue, Birjand, 9713643138 Iran

**Keywords:** Aluminum phosphide, N-acetyl cysteine, Pesticide, Rice tablet

## Abstract

**Background:**

Aluminum phosphide (AlP) is a very effective indoor and outdoor pesticide. We investigated the effects of N-acetyl cysteine (NAC) on the survival time, hemodynamics, and cardiac biochemical parameters at various time intervals in some cases of AlP poisoning.

**Methods:**

This research was a case–control study to evaluate 63 AlP poisoned patients during 2010–2012. Patients with cardiovascular complications of AlP to be treated with intravenous NAC plus conventional treatment were considered as the case group and compared with patients who did not receive NAC. NAC infusion was administered to the case group at 300 mg/kg for 20 h. The data gathered included age, sex, heart rate, Systolic blood pressure (SBP), creatine phosphokinase (CPK), creatine kinase MB (CK-MB), and ECG at the admission time and 12, 18, and 24 h after admission. Analysis of repeated measures was performed to check the variability of parameters over time.

**Results:**

The mean ages in the case and control groups were 26.65 ± 1.06 (19–37 years) and 28.39 ± 1.11 (18–37 years), respectively (P = 0.266). Most of the patients were female (56.5%). CK-MB means were significantly different between the two groups, but no differences between the other variables were observed. Also, CK-MB, CPK, heart rate, and systolic blood pressure means became significantly different over time (0, 12, 18, and 24 h) in both groups (P < 0.001). NAC prevented sharp heart rate fluctuations in AlP patients in the case group. Regarding the outcomes, 17 patients died (10 patients in the control and 7 patients in the case groups). No side-effects of NAC were observed.

**Conclusion:**

Our patients could be managed by the positive role of NAC as the biochemical index of cardiotoxicity was found to elevate in both the case and control groups. Therefore, for the management protocol optimization, NAC evaluation should be done in further cases.

## Background

Aluminum phosphide (AlP) is a very effective indoor and outdoor pesticide used in some developing countries (Mehrpour and Singh 2010). In Iran, it is also called rice tablet, which is mainly used to protect rice and grains during storage. The main mechanisms of toxicity are inhibition of cytochrome oxidase c, as well as oxidative stress (Mehrpour et al. 2009, 2014a). After contacting with moisture, AlP releases phosphine gas, which is a lethal poison (Mehrpour et al. 2012). The mortality rates caused by AlP poisoning have been demonstrated as high as 70–100% in various studies (Mehrpour et al. 2012). The main cause of death is a refractory cardiogenic shock (Mehrpour et al. 2011, 2014b). Moreover, other contributing factors include severe hypotension and severe and refractory metabolic acidosis. Since the cardiovascular system is the main target of this poison, various electrocardiographic changes including dysrhythmias may occur by this fatal poisoning.

NAC is an important antioxidant and a cytoprotective agent that replenishes intracellular glutathione. In animal studies, NAC has been shown to have a protective role against cardiovascular complications by protecting heart cells from the oxidative stress induced by phosphine (Shakeri and Mehrpour 2014). Therefore, we investigated the effects of NAC on hemodynamics and cardiac biochemical parameters of AlP poisoning cases at various time intervals as well as during the survival time and compared them with those of the control group.

## Methods

This research was a case–control study conducted in Baharloo Teaching Hospital, a referral poisoning center in Tehran, Iran during 2010–2012 (Mehrpour and Abdollahi 2012). AlP-poisoned patients were included in the study during the study period. The patients without any symptoms and signs for 12 h and unreliable history and those who died during the first 24 h were excluded from the study. The treatment group (case group) received intravenous NAC plus a conventional treatment. The control group consisted of the patients undergoing only conventional treatment for AlP poisoning. The conventional treatment as our local guideline was administered to both groups included lavage with KMnO_4_, magnesium sulphate (1 g initially followed by 1 g every 6 h), 10% calcium gluconate (1 g initially followed by 1 g every 6 h), hydrocortisone (200 mg initially followed by 200 mg every 6 h), vitamin C (1000 mg every 12 h via slow intravenous infusion), vitamin E (400 units intramuscularly) NaHCO3 administration for treatment of acidosis (Mehrpour et al. 2008; Oghabian and Mehrpour 2016). NAC infusion was administered to the mentioned group at 300 mg/kg for 20 h. The outcomes were considered for changes in the hemodynamic and cardiac biochemical parameters at the survival time. The patients were also evaluated based on NAC side-effects.

The data gathered included age, sex, heart rate, blood pressure, creatine phosphokinase (CPK), creatine kinase-MB fraction (CK-MB), and ECG at the time of admission and 12, 18, and, 24 h after admission.

The patients were transferred to the Intensive Care Unit (ICU) in case they had an arterial pH less than 7.34 besides cardiovascular symptoms. The data were recorded on a checklist. IBM SPSS 22 was used for the statistical analysis. The data were presented as mean ± SE. Chi Square or Fisher’s exact test was used to compare the two groups. Also, analysis of repeated measures was performed to compare the means between and among the groups at different evaluation times. Figures show the estimated marginal means in each time for both groups. The Estimated Marginal Means are the mean response for each factor, adjusted for any other variables in the model. It means that the Estimated Marginal Means adjust for the covariate by reporting the means of response variable for each level of the factor at the mean value of the covariate. A P value less than 0.05 was considered as significant. An informed consent was obtained from alert patients or their first-degree families or relatives. The study protocol was approved by the ethical committee of Tehran University of Medical Sciences, Tehran, Iran (No. 345).

## Results

63 Patients with AlP poisoning (caused by oral consumption) were admitted during the study period. The data of 46 patients (23 patients in each group) were analyzed in the study since 17 patients died and were excluded from the study.

The mean ages in the case and control groups were 26.65 ± 1.06 (19–37 years) and 28.39 ± 1.11 years (18–37 years), respectively (P = 0.26). Most of the patients were female (56.5%) (69.6 and 43.5% in the case and control groups, respectively).

The analysis of variance and repeated measures showed that CK-MB means were significantly different between the two groups. However, no significant differences were seen between the groups based on the other variables. Also, CK-MB, CPK, heart rate, and systolic blood pressure means were significantly different over time (0, 12, 18, and 24 h) in the case and control groups. The results revealed that CK-MB means were significantly different after 12, 18, and 24 h of admission in comparison with the admission times in both groups (P < 0.001). Also, there were significant differences between CPK means after 18 and 24 h of admission compared to the admission times in both groups (P < 0.001). Heart rate means were different 18 h after admission compared to the admission time in the control group (P < 0.001). There were significant differences between systolic blood pressure means after admission times compared with the admission time in NAC group (P < 0.001). Different means were obtained between 12 and 18 h after admission and at the admission time in the control group (P < 0.001) (Table 1).

**Table 1:** Characteristics of variables in different times in two groups

Variables	Group	Admission time	12 h after admission	18 h after admission	24 h after admission
		Mean (SE)	Mean (SE)	Mean (SE)	Mean (SE)
CK-MB	Control	20.45 (1.1)	40.54 (0.9)*	51.45 (1.7)*	70.09 (2.6)*
	Case (NAC)	23.69 (1.8)	40.53 (1.9)*	52.30 (2.3)*	68.07 (2.2)*
	P value	0.54	0.76	0.11	0.21
CPK	Control	320.90 (38.6)	344.90 (23.5)	726.36 (44.1)*	1182.81 (114.9)*
	Case (NAC)	358.15 (48.8)	367.69 (30.2)	726.69 (68.2)*	1004.69 (172.5)*
	P value	0.82	0.51	0.34	0.31
HR	Control	80.36 (3.9)	73.45 (4.7)	69.63 (5.1)*	75.81 (4.0)
	Case (NAC)	82.76 (3.1)	77.69 (6.1)	78.46 (4.6)	77.76 (3.7)
	P value	0.88	0.75	0.45	0.56
SBP	Control	106.63 (2.4)	98.81 (2.5)*	93.18 (1.2)*	96.54 (1.2)
	Case (NAC)	100.15 (3.9)	93.30 (1.5)*	94.38 (4.1)*	102.61 (2.7)*
	P value	0.23	0.94	0.55	0.32

*CK-MB* creatine kinase-MB fraction, *CPK* creatine phosphokinase, *HR* heart rate, *SBP* systolic blood pressure case group received NAC beside conventional therapy

* Significantly different with admission time in each group

At the time of admission, sinus tachycardia, sinus bradycardia, PVC, QRS widening, ST-elevation MI were observed in 13.0, 8.7, 4.3, 8.7, and 8.7% in the control group respectively. In the case group, sinus tachycardia, sinus bradycardia, PVC, widening of QRS, and ST-elevation MI were discovered in 17.4, 4.3, 4.3, 8.7, and 4.3% of the patients, respectively. 12 h after admission, Sinus tachycardia, sinus bradycardia, PVC, widening of QRS, ST-elevation MI, and AF were seen in 17.4, 4.3, 4.3, 8.7, 8.7, and 4.3% of the patients in the case group, respectively. After 18 h, sinus bradycardia, PAC, widening of QRS, ST-elevation MI, VT, and VF were observed in 8.7, 4.3, 4.3, 8.7, 13.0, and 4.3% of the case group, respectively, while 2 patients died. The evaluations after 24 h of admission indicated that sinus tachycardia, sinus bradycardia, PAC, widening of QRS, ST-elevation MI, AF, and VT had occurred in 4.3, 6.3, 4.3, 4.3, 13.0, 4.3, and 4.3% of patients in the case group, respectively. There were no significant differences between the two groups (P value >0.05) (Table 2).

**Table 2:** Distribution of ECG changes in different times

ECG changes	Admission time		12 h		18 h		24 h	
	Case (%)	Control (%)	Case (%)	Control (%)	Case (%)	Control (%)	Case (%)	Control (%)
No evidence	56.5	60.9	39.1	47.8	34.8	43.5	26.1	30.4
Sinus tachycardia	13.0	17.4	17.4	17.4	8.7	8.7	4.3	4.3
Sinus bradycardia	8.7	4.3	4.3	NAD	NAD	NAD	4.3	4.3
PVC	4.3	4.3	4.3	NAD	NAD	NAD	NAD	NAD
PAC	NAD	NAD	NAD	NAD	4.3	4.3	4.3	NAD
QRS widening	8.7	8.7	8.7	8.7	4.3	NAD	4.3	NAD
ST. elevation	8.7	4.3	8.7	NAD	8.7	NAD	13.0	NAD
A.F.	NAD	NAD	4.3	NAD	NAD	NAD	4.3	NAD
V.T	NAD	NAD	NAD	NAD	13.0	21.7	4.3	NAD
V.F.	NAD	NAD	NAD	NAD	4.3	4.3	NAD	4.3

*NAD* no abnormality detected

**Fig. 1:**
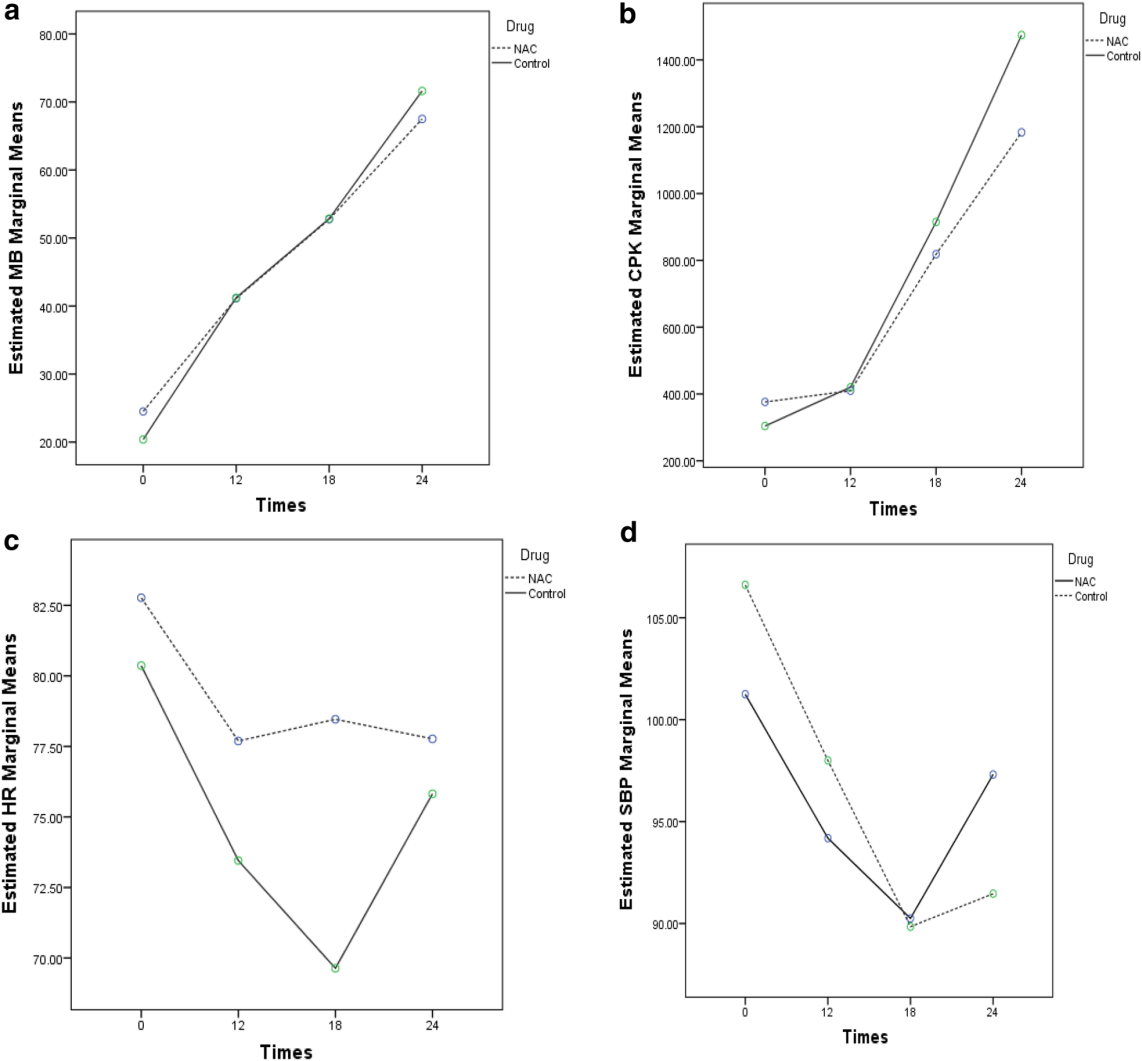
Estimated marginal means in two groups over time

A sudden drop in the heart rates of patients in the control group was observed after 12 and 18 h of admission and then they increased (Fig. 1c). However, using NAC in the case group could prevent sharp fluctuations in the heart rates in AlP patients (Fig. 1e). Moreover, a sudden decrease in the systolic blood pressures of the patients who had received NAC and those of the control group in receiving it in a similar way was observed, while the systolic blood pressures rose sharply to their normal values in the patients who had got NAC for their treatments between 18 and 24 h after admission (Fig. 1d). Regarding the outcomes, 17 patients died [10 patients in the control and 7 patients in the treatment (case) groups]. No side-effects of NAC were observed.

## Discussion

The generation of superoxide radicals and cellular peroxides is resulted from the inhibitory effects of phosphine on mitochondrial cytochrome-C oxidase. Lipid peroxidation and other oxidant mechanisms subsequently lead to cellular injury. It has been reported that the toxins generated by AlP ingestion leads to a profound circulatory collapse, as the major lethal consequence that directly affects cardiac myocytes and causes adrenal gland damage and fluid loss (Mehrpour et al. 2009, 2012, 2014a; Mehrpour and Singh 2010).

So far, supportive therapy is the main treatment for such patients since there is no specific antidote to stop AlP poisoning (Mostafazadeh and Farzaneh 2012).

Treatment of cardiogenic shock as one of the main causes of death in AlP poisoning may reduce the relevant mortality (Mehrpour et al. 2011, 2012, 2014b). Intra-aortic Balloon Pump **(**IABP) (Mehrpour et al. 2014b), glucagon (Oghabian and Mehrpour 2016), and digoxin (Mehrpour et al. 2011) are used to manage cardiogenic shock in some treatments. IABP has been shown to be another excellent treatment for AlP poisoning by previous studies. Moreover, in severe cases of AlP poisoning, IABP addition to the treatment protocol has been strongly recommended by some researchers besides using Extracorporeal Membrane Oxygenation (ECMO) (Hassanian-Moghaddam et al. 2016).

Acidosis can be reversed through improved uptake of myocyte carbohydrate (Hassanian-Moghaddam and Zamani 2016). Also, the use of hyperinsulinemia/euglycemia (HIE) as another useful treatment improves inotropy and peripheral vascular resistance (Hassanian-Moghaddam and Zamani 2016).

Furthermore, coconut oil, which is believed to inhibit phosphine gas release from AlP due to the physicochemical properties of aluminium phosphide and its non-miscibility with fat (Shadnia et al. 2005) has been suggested for gastric lavage in acute AlP poisoning by some researchers (Shadnia et al. 2005).

Oxidative stress is one of the main mechanisms of AlP to induce toxicity (Mehrpour et al. 2012, 2014a). Vitamins C and E and N-acetylcysteine (Oghabian and Mehrpour 2016) as antioxidant agents have been shown by the previous studies to have significant benefits in AlP poisoning management. Recently, via a combination of the mentioned antioxidant agents and intravenous glucagon and digoxin, AlP poisoning has been successfully reported to be treated by Oghabian and Mehrpour (2016). Meanwhile, that oxidative stress is believed by Abdollahi et al. (2004) to be one of the main mechanisms of AlP toxicity, which is somewhat similar to those of organophosphate (OP) compounds (Abdollahi et al. 2004).

Also, glutathione as a main antioxidant defense is reduced by AlP. Actually, changes in glucose metabolism were associated with AlP and OP that cause a toxic stress in a similar way (Rahimi and Abdollahi 2007; Nath et al. 2011). Antioxidant agents seem to usefully reduce the toxicity. Cellular glutathione and magnesium have been shown to be replenished in rats (Hsu et al. 2000, 2002) and humans (Chugh et al. 1997) with the help of N-acetylcysteine, which has antioxidant properties through different studies. Myocardial oxidative injury in rats exposed to AlP has been shown to be reduced by N-acetylcysteine, while increasing their survival times (Bogle et al. 2006).

In this study, N-acetylcysteine was examined for reducing oxidative stress and AlP causing cardiotoxicity. In these patients, cardiogenic shock and cardiotoxicity are the most important causes of mortality. Various types of cardiac dysrhythmia, such as VT (17.4%), sinus tachycardia (10.87%), VF (8.7%), ST elevation (8.7%), widening of QRS (8.7%), sinus bradycardia (6.5%), PAC (6.5%), PVC (2.2%), and AF (2.2%) were discovered in this research. No significant difference was found between the groups though the case group was associated with a lower rate of dysrhythmia. In cases of AlP poisoning various types of cardiac events or dysrhythmia may be happened such as sinus tachycardia. VF, VT, ST elevation, widening of QRS, sinus bradycardia, PAC, PVC, and AF. Of them VF and VT seems to be terminal cardiac dysrhythmia before death (Mehrpour et al. 2012).

In the previous studies, ECG changes including ST-T changes, AV conduction disturbances, bundle branch blocks, supraventricular and ventricular tachycardia, and atrial fibrillation have been precisely investigated (Jain Sm et al. 1985; Chugh et al. 1989, 1991; Soltaninejad et al. 2012).

In their study, Chugh et al. found 50% of ECG abnormality incidence in AlP poisoning cases. Nearly, equal frequencies of ischemic pattern, conduction disturbances, and dysrhythmias were discovered by them. In their study, varied electrical alternates, sino-atrial blocks, and early repolarization and bradycardia-tachycardia syndromes were observed, while no ECG abnormality effects on mortality were found (Jain Sm et al. 1985). 45% of dysrhythmia cases were discovered by Soltaninejad et al. 20, 35, 45, and 45% of the intervals, prolonged QTc, Bundle Branch Block (BBB), and ST-segment elevation were seen, respectively (Soltaninejad et al. 2012).

Myocardial damage caused by AlP poisoning was also confirmed by biochemical biomarkers of cardiac muscle injury, such as CPK, CK-MB, and Troponin-T (Soltaninejad et al. 2012). Myocardial damage can be traced by the many folds of CPK-MB and LDH elevations in AlP poisoning (Shah et al. 2009; Anand et al. 2012) though elevation of CPK levels without any changes in CPK-MB fraction was reported by Duenas et al. (1999). Contrary to our finding, CPK levels and CPK/CPK-MB ratio were not found to be reliable cardiac injury markers in AlP poisoning by Soltaninejad et al. (2012). However, normal CPK levels at hospital admission were seen in another case report by Nayyar and Nair (2009). Though controversial in some AlP poisoning case reports, cardiac muscle injury has been clinically and laboratorially indicated by the changes in CPK and CK-MB levels. In some case reports, these markers led to normal serum levels after AlP poisoning (Bogle et al. 2006; Nayyar and Nair 2009). Nevertheless, myocardial injury caused by a rise in their levels was indicated in other reports (Akkaoui et al. 2007; Kaushik et al. 2007; Shah et al. 2009). Despite ECG changes in acute AlP poisoning, inconsistent reports of normal and abnormal CPK-MB levels are found (Kaushik et al. 2007; Soltaninejad et al. 2009). In addition, in a review article by Karami & Mohajeri, it was concluded that the normal levels of these enzymes cannot disprove cardiotoxicity though their elevated levels can corroborate myocardial damage (Karami-Mohajeri et al. 2013).

For the first time, CPK and CK-MB repeated measurements were used in this study. Also, the four cardiac indices of CPK, CK-MB, SBP, and heart rate were evaluated and compared in the case and control groups. Myocardial damage during AlP poisoning was confirmed by CPK and CK-MB enhancements in both groups with the passage of time. However, lower rates of CPK and CK-MB, especially after 12 and 18 h, respectively, and bradycardia deceleration in SBP over time were revealed in the case group receiving NAC. AlP poisoning treatment with antioxidants, especially NAC in our study was the same result obtained by other investigations (Duenas et al. 1999). Meanwhile, significantly lower rates of intubation, ventilation, and mortality and shorter duration of hospitalization were found in AlP-poisoned patients treated with NAC compared to the controls in the previous study (Tehrani et al. 2013).

Some limitations in our study included exclusion of patients who died within the first 24 h of admission and inability to analyze the different variables between the two groups based on alive or dead patients due to the small number of dead patients in both groups. Nonetheless, reduced rate of mortality in AlP patients was clearly found to be resulted from NAC effect on some cardiac variables, such as heart rate and SBP in this study.

## Conclusion

Our patients could be managed by the positive role of NAC as the biochemical index of cardiotoxicity was found to elevate in both the case and control groups. Therefore, for the management protocol optimization, NAC evaluation should be done in further cases.

## Abbreviations

Alp: aluminum phosphide
NAC: N-acetyl cysteine
CK-MB: creatine kinase MB
ECG: electrocardiography
SBP: systolic blood pressure
CPK: creatine phosphokinase
ICU: Intensive Care Unit
PVC: premature ventricular contraction
MI: myocardial infarction
AF: atrial fibrillation
VT: ventricular tachycardia
VF: ventricular fibrillation
HIE: hyperinsulinemia/euglycemia
OP: organophosphate
